# *Merlin* is required for coordinating proliferation of two stem cell lineages in the *Drosophila* testis

**DOI:** 10.1038/s41598-017-02768-z

**Published:** 2017-05-31

**Authors:** Mayu Inaba, Dorothy R. Sorenson, Matt Kortus, Viktoria Salzmann, Yukiko M. Yamashita

**Affiliations:** 1 Life Sciences Institute, Center for Stem Cell Biology, Ann Arbor, Michigan United States; 2 Department of Cell and Developmental Biology, School of Medicine, Ann Arbor, Michigan United States; 3 Howard Hughes Medical Institute, University of Michigan, Ann Arbor, Michigan United States; 4 263 Farmington Ave. E6053, Department of Cell Biology, UConn Health, Farmington, CT 06030 USA

## Abstract

Although the mechanisms that balance self-renewal and differentiation of a stem cell lineage have been extensively studied, it remains poorly understood how tissues that contain multiple stem cell lineages maintain balanced proliferation among distinct lineages: when stem cells of a particular lineage proliferate, how do the other lineages respond to maintain the correct ratio of cells among linages? Here, we show that *Merlin* (*Mer*), a homolog of the human tumor suppressor *neurofibromatosis 2*, is required to coordinate proliferation of germline stem cells (GSCs) and somatic cyst stem cells (CySCs) in the *Drosophila* testis. *Mer* mutant CySCs fail to coordinate their proliferation with that of GSCs in multiple settings, and can be triggered to undergo tumorous overproliferation. *Mer* executes its function by stabilizing adherens junctions. Given the known role of *Mer* in contact-dependent inhibition of proliferation, we propose that the proliferation of CySCs are regulated by crowdedness, or confluency, of cells in their lineage with respect to that of germline, thereby coordinating the proliferation of two lineages.

## Introduction

The balance between stem cell self-renewal and differentiation is critical for maintenance of functional tissues. Asymmetric stem cell division balances the number of stem cells and differentiated cells of a particular lineage^1, 2^. However, tissues that contain multiple stem cell lineages must further coordinate the proliferation rates among distinct lineages such that the correct ratio of all cell types is maintained within the tissue. The lack of coordination among multiple stem cell lineages may cause unbalanced proliferation of a certain lineage with respect to others, leading to disruption of tissue architecture. Such disruption can be a triggering event for more complex pathologies, including tumorigenesis and tissue degeneration. Indeed, recent findings reveal the presence of coordination between multiple stem cells that share the niche^3, 4^. However, the mechanisms by which proliferation of multiple stem cell lineages is coordinated remain poorly explored.

**Figure 1:**
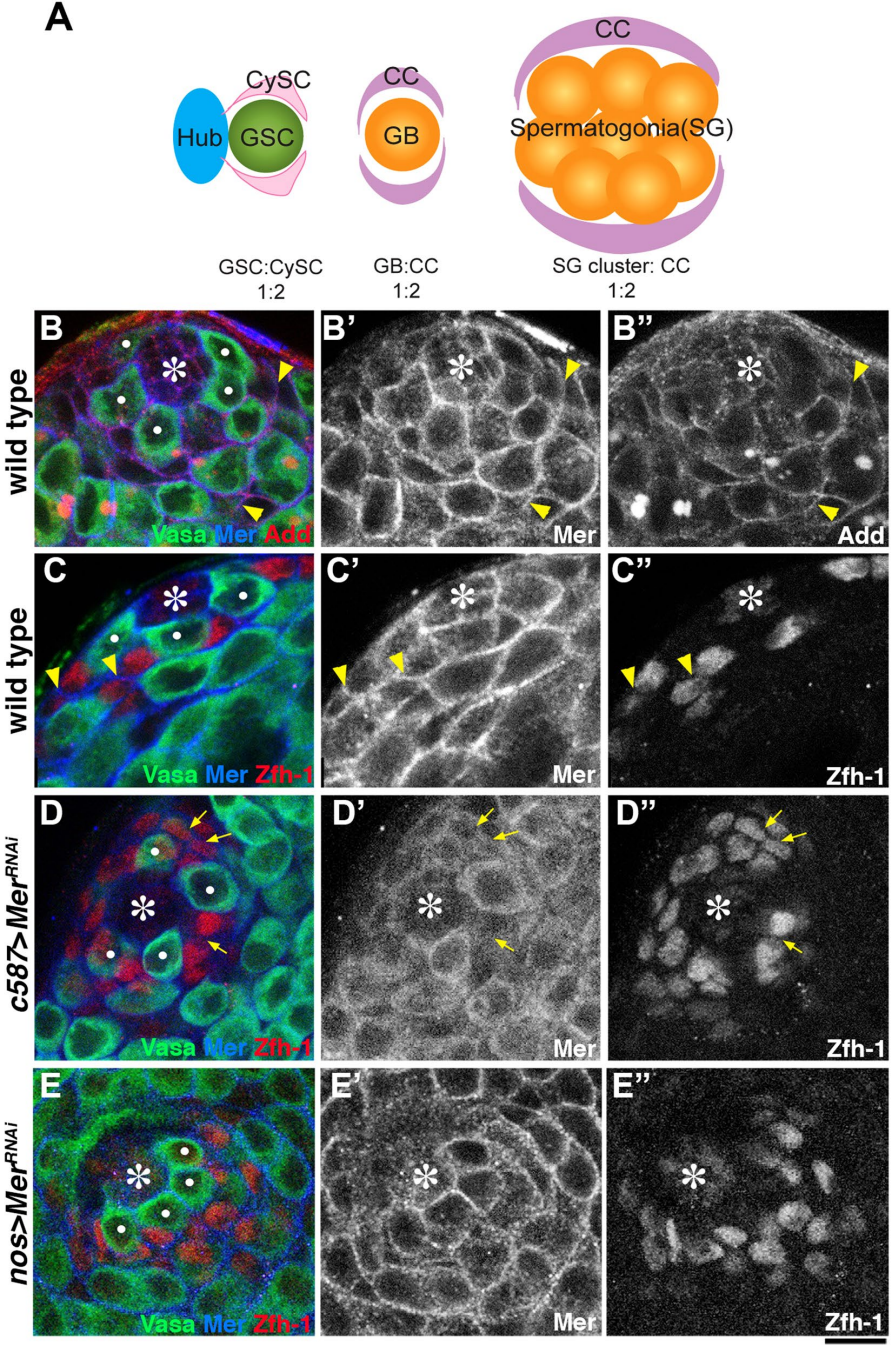
*Mer* protein localizes to the surfaces of CySCs and CCs. (**A**) Diagram of the *Drosophila* testicular stem cell niche. GSCs and CySCs are attached to the hub cells, where each GSC is encapsulated by a pair of CySCs. GB, the differentiating daughter of a GSC, which will become spermatogonia (SGs), is encapsulated by a pair of CCs generated by CySC divisions. (**B** and **C**) The wild-type testis apical tip shows *Mer* protein localization on the cell surface (arrowheads). The pseudocolor of immunofluorescent staining is shown in the colored text. GSCs are indicated by white dots. Bar, 10 µm. Hub (*). (**D**) RNAi-mediated knockdown of *Mer* in the CySC lineage (*c587* > *Mer* ^*RNAi*^). Residual cytoplasmic staining can be seen in the germline. (**E**) RNAi-mediated *Mer* knockdown in the germline (*nos* > *Mer* ^*RNAi*^).

*Drosophila* testis contains two stem cell populations, germline stem cells (GSCs) and somatic cyst stem cells (CySCs), which cohere to and regulate each other. Both stem cell types attach to hub cells at the apical tip of the testis (Fig. 1A)^5^. Each GSC is encapsulated by a pair of CySCs, whereas the differentiating daughter of the GSC, gonialblast (GB), is encapsulated by a pair of cyst cells (CCs; differentiating daughters of CySCs). Encapsulation of germ cells by somatic cells is essential for GSC maintenance and germ cell differentiation^6^. These relationships between germline and somatic lineages create the necessity for coordinated proliferation between GSCs and CySCs. Indeed, we have shown that mitotic indices of GSCs and CySCs is 1:2 in ratio^7^, indicating the presence of mechanism(s) that coordinate their proliferation. However, underlying mechanisms of their coordination remain unknown.

*Merlin* (*Mer*) is a homolog of the *neurofibromatosis 2* (*Nf2*) tumor suppressor gene, which is mutated in a rare cancer neurofibromatosis type 2, characterized by central and peripheral nervous system tumors derived from Schwann cells^8, 9^. *Mer*’s function to regulate cell proliferation is conserved through evolution^10, 11^. *Mer* encodes a protein closely related to ezrin, radixin, and moesin (ERM) proteins, and functions to stabilize the membrane-cytoskeleton interface. In cell culture models, *Mer* has been shown to function in contact-dependent inhibition of proliferation (‘contact inhibition’ in short) through stabilization of adherens junctions and regulation of signaling events at the cell cortex^9, 12^. Contact inhibition is characterized by halted proliferation of cells in culture, when cells reach confluence. Transformed cells override contact inhibition and maintain proliferation, yielding a multilayered stack of cells. Contact inhibition is triggered by cell-cell contact, where the adherens junction plays a key role in sensing confluency and inhibiting further proliferation. In mouse models, *Mer* is required for tissue homeostasis in the liver, where *Mer* mutation leads to overgrowth of the tissue^13, 14^. However, it is not well understood how the contact inhibition mechanism elucidated through cell culture models applies to *in vivo* settings, where multiple cell types are organized into complex tissue architecture.

Here we show that *Mer* is required to prevent excess proliferation of CySCs in relation to GSCs in the *Drosophila* testis. In *Mer* mutant testes, CySCs’ proliferation is not well coordinated with GSCs, leading to an increase in the number of CCs. This lack of coordination is further highlighted when CySC proliferation is stimulated by expression of bone morphogenetic protein ligand decapentaplegic (Dpp). Although Dpp’s activity to stimulate CySC proliferation is normally masked by the *Mer*-dependent mechanism that suppresses excess CySC proliferation, the combination of Dpp stimulation and *Mer* mutation leads to unlimited proliferation of CySCs/CCs. In another setting, in which germ cells are depleted, wild type CySCs cease proliferation, whereas *Mer* mutant CySCs continue to proliferate, demonstrating *Mer*’s role to suppress CySC proliferation in the absence of germ cell proliferation. We further provide evidence that *Mer*’s function to regulate CySC/CC proliferation involves E-cadherin. We propose that *Mer* regulates coordination of proliferation between GSCs and CySCs by limiting excess proliferation of CySCs via the mechanism equivalent to contact inhibition. Our work provides insights into how tissues composed of multiple cell types might achieve coordinated proliferation rates to maintain tissue homeostasis.

## Results

### *Mer* protein localizes to the cell cortex of somatic CySCs and CCs at the apical tip of the *Drosophila* testis

Using the anti-Mer antibody described previously^15^, we found that Mer protein localizes to the cell cortex at the apical tip of the *Drosophila* testis (Fig. 1B and C). GSCs were identified by expression of Vasa, a germline-specific protein, and attachment to the hub. CySCs and their recent daughters were identified by the expression of the transcriptional repressor Zfh-1^16^. By using these markers and anti-Mer antibody, Mer was clearly observed on the plasma membrane of cells in the apical tip of the testes. Because germ cells and somatic cells closely associate with each other, it is impossible to distinguish whether Mer protein localizes to the germ cell cortex or the somatic cell cortex. In some cases, however, membrane localization was observed between two somatic cells (Fig. 1B and C, arrowheads), suggesting that Mer protein localizes to the somatic cell cortex. This notion was further confirmed by lineage-specific, RNAi-mediated knockdown of *Mer*. When *Mer* was knocked down in the CySC lineage (*c587-gal4* > *UAS-Mer* ^*RNAi*^), the membrane localization of Mer protein was almost completely abolished (Fig. 1D). In contrast, when *Mer* was knocked down in the germline (*nos-gal4* > *UAS-Mer* ^*RNAi*^), the cortical localization of Mer protein remained intact (Fig. 1E), suggesting that the majority of observed cortical localization of Mer protein is due to its expression in the CySC lineage. Furthermore, the phenotype of *Mer* ^*RNAi*^ in the CySC lineage recapitulates the loss-of-function allele (*Mer* ^*ts1*^, see below), suggesting that *Mer* mainly functions in the CySC lineage in the *Drosophila* testis.

### *Mer* is required to suppress excess numbers of cyst cells

To examine the function of *Mer* in the *Drosophila* testis, we used a temperature sensitive, loss-of-function allele of *Mer* (*Mer* ^*ts1*^) and RNAi-mediated knockdown of *Mer* (*Mer* ^*RNAi*^) in the CySC lineage (*c587-gal4* > *UAS-Mer* ^*RNAi*^). To assess possible changes in the GSC/CySC/CC populations, we used above-mentioned Vasa and Zfh-1, as well as the transcription factor Traffic jam (Tj): Tj marks CySCs and CCs at early stages of differentiation^17^, a slightly broader range of CCs compared to the population marked by Zfh-1.

**Figure 2:**
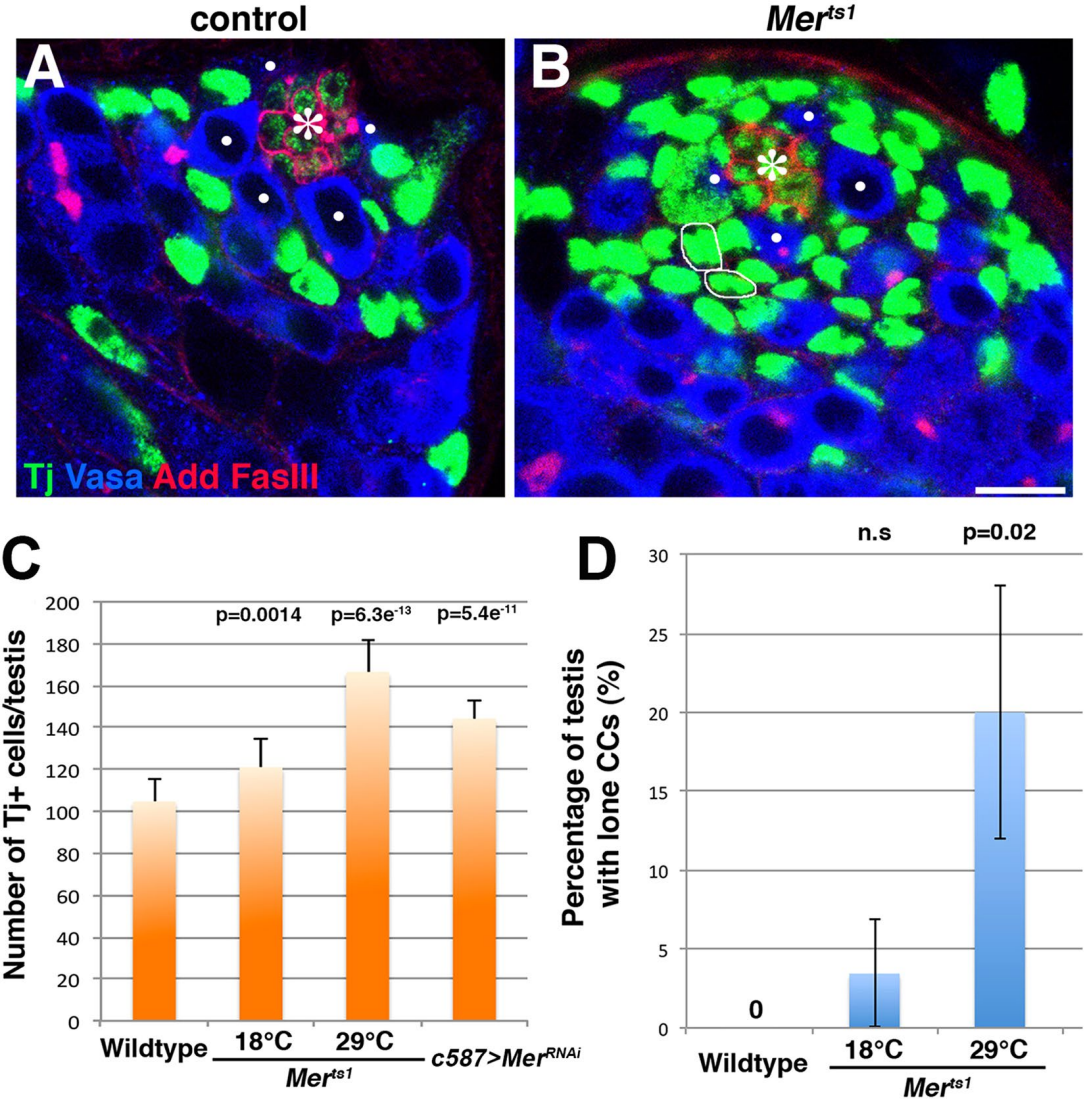
CCs are increased in number upon loss of *Mer* function.(**A** and **B**) The testis apical tip stained for Tj (green), Vasa (blue), adducin-like (Add), and fasciclin III (Fas III, red) in a control (**A**) and (*Mer* ^*ts1*^) mutant (**B**) testis. Circles in (**B**) indicate Tj^+^ cells without clear association with germ cells (“lone CCs”). GSCs are indicated by white dots. Hub (*). Bar, 10 µm. (**C**) The number of Tj^+^ cells/testis in control, *Mer* ^*ts1*^, and *c587* > *Mer* ^*RNAi*^ testes. Data are expressed as the mean ± SD, and *p* values were obtained using the Student’s *t* test (two-tailed) by comparing to wild type. N = 15 testes for data point. (**D**) The frequency of testes containing lone CCs in control and *Mer* mutants. N ≥ 40 testes for data point.

In wild type/control testes, due to coordinated proliferation of GSCs and CySCs^7^, we barely observed excess germ cells that were not associated with any CCs, or excess CCs that were not associated with any germ cells. Accordingly, CCs positive for Tj were well interspersed among germ cells in control testis (Fig. 2A). In contrast, we found that *Mer* ^*ts1*^ or *Mer* ^*RNAi*^ testes contained higher number of CCs (~140 Tj^+^ cells in *Mer* ^*RNAi*^ testes and ~160 Tj^+^ cells in *Mer* ^*ts1*^ testes compared to ~100 Tj^+^ cells in control testes, Fig. 2B and C), and we often observed excess CCs that did not apparently touch any germ cells (Fig. 2B, white circle, and D). Excess CCs were not due to increased CySC number (Zfh-1^+^ cell number) or division rate (Supplementary Figure S1A). Also, both in wild type and *Mer* mutant testes, the only somatic cells observed in mitosis were CySCs (Supplementary Figure S1B, C, 100% for N = 48 for *Mer* ^*ts1*^ control, N = 66 for *Mer* ^*ts1*^ at 29 °C, N = 57 for *c587-gal4* control, N = 114 *c587-gal4* > *UAS-Mer* ^*RNAi*^), excluding the possibility that *Mer* mutant CCs divide to increase in number. In addition, the increase of Tj^+^ cells in *Mer* mutant testes was unlikely due to a defect in CC differentiation, since we observed normal expression patterns of Eyes absent (Eya), a marker for differentiated CCs (Supplementary Figure S2).

It is well established that *Mer* functions via the regulation of the Hippo pathway in many cell types examined. *Mer* acts upstream of the Hippo pathway to ultimately downregulate the function of the pro-proliferative transcription factor Yki, leading to suppression of cell proliferation^8, 10, 18, 19^. Overexpression of *yki* can mimic the loss of function of Hippo pathway components. However, we found no significant changes in the number of GSCs or Tj^+^ CCs upon overexpression of wild-type *yki* or a constitutive-active form of *yki* in the CySC lineage (Supplementary Figure S3), suggesting that *Mer* functions independently of *yki* to regulate the number of CCs. RNAi-mediated knockdown of *hippo* using independent RNAi lines had no effect on Tj^+^ CC number, either (Supplementary Figure S3). These results suggest that the CySC proliferation by *Mer* is unlikely mediated by the canonical Hippo pathway. Hippo-independent function of *Mer* is reminiscent of *Nf2* function reported in mouse liver^13^ and culture cells^20^. It was recently shown that Hippo pathway is active in CC lineage and that *hippo* mutant CySCs outcompete wild type CySCs in the niche^21^. This suggests that CySCs’ proliferation within its own lineage is under the regulation of Hippo pathway, whereas CySCs’ coordinated proliferation in relation to germline is regulated by *Mer*, independent of Hippo pathway. How these two pathways may together regulate overall CySC proliferation awaits future investigation.

### Adherens junction between CCs is compromised in *Mer* mutant testes

**Figure 3:**
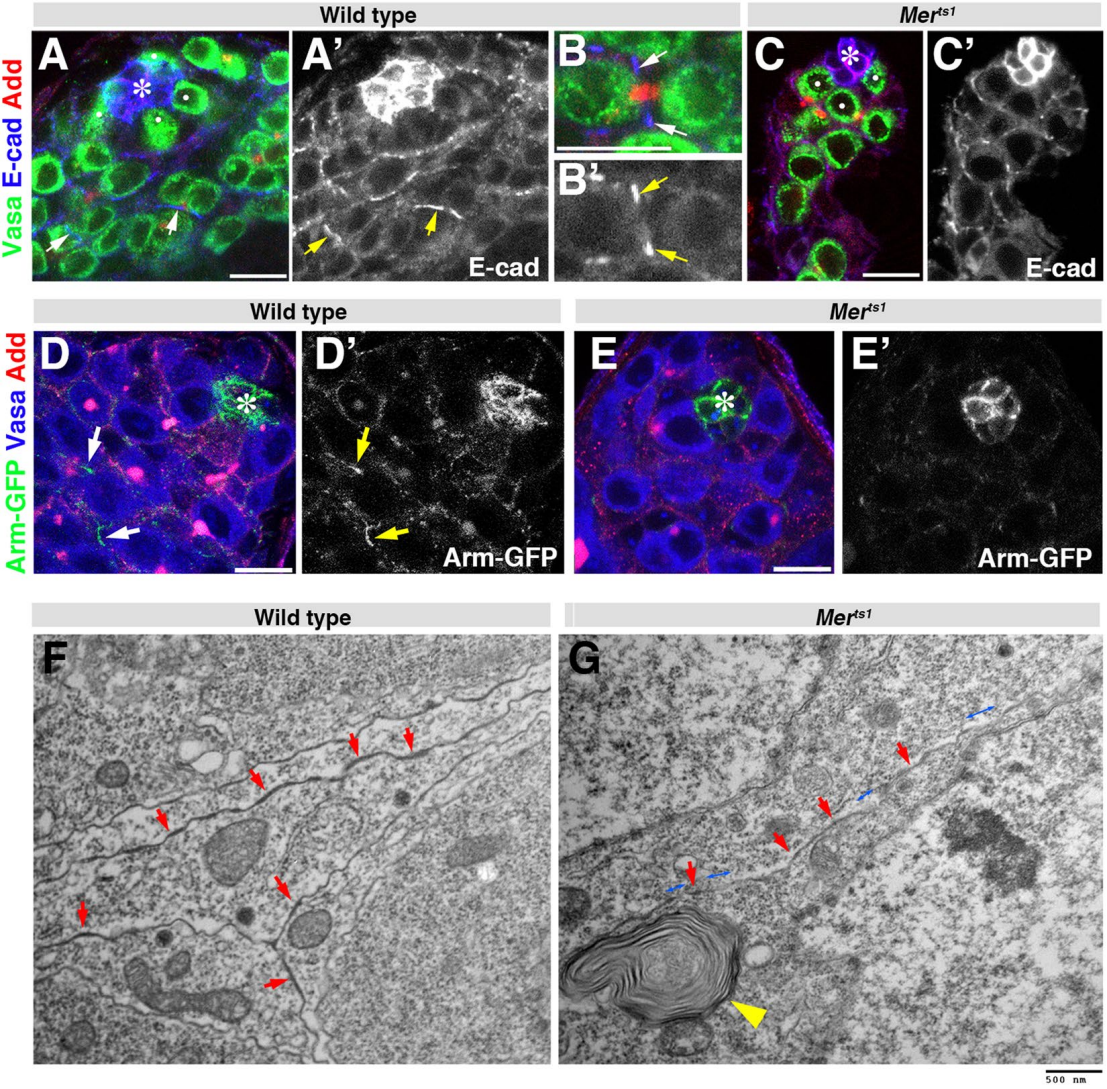
*Mer* is required for stability of adherens junction between CCs. (**A**–**C**) Apical tip of a wild-type (**A** and **B**) and *Mer* ^*ts1*^ mutant (**C**) testis stained for E-cadherin (Blue), Vasa (Green), and Add (Red). GSCs are indicated by white dots. Hub (*). Bar, 10 µm. (**D** and **E**) Apical tip of wild-type (**D**) and *Mer* ^*ts1*^ mutant (**E**) testis visualized for Arm-GFP (Green), Vasa (Blue), and Add (Red). (**F** and **G**) Transmission electron microscopy showing junctions between two somatic cells in a wild-type (**F**) and *Mer* ^*ts1*^ (**G**) testis. Arrows indicate cell-cell junctions. Blue, double-headed arrows in (**F**) indicate gaps in cell-cell junction structure. We noted that *Mer* ^*ts1*^ mutant somatic cells have numerous multilamellar bodies (yellow arrowhead), although its meaning is currently unclear. Bar, 500 nm.

Because the Hippo pathway is apparently not involved in *Mer*-mediated regulation of CC number, we sought other mechanisms that could explain *Mer* mutant phenotypes in the *Drosophila* testis. In cultured cells, *Mer/Nf2* plays a role in the contact-dependent inhibition of proliferation via its ability to regulate the adherens junctions^8, 22, 23^. Therefore, we examined possible effect(s) of *Mer* ^*ts1*^ mutation on adherens junctions. In wild-type testes, we observed that E-cadherin, a major component of adherens junctions, localized to the cell-cell junction between CCs (Fig. 3A,B, arrows), in addition to its well-characterized localization to the hub-GSC and hub-CySC interface (Fig. 3A, asterisk), where E-cadherin supports anchorage of GSCs and CySCs to the hub^24–27^. In *Mer* ^*ts1*^ mutants, E-cadherin localization at hub cells was not visibly affected (Fig. 3C, asterisk). However, its localization between CCs was less prominent, compared to wild type (Fig. 3C). Similarly, GFP-Armadillo (Arm, β-catenin), another component of adherens junctions, was observed between somatic cells in control (Fig. 3D) but not in *Mer* ^*ts1*^ mutant (Fig. 3E), suggesting that *Mer* is required for adherens junction stability between CCs.

We further characterized the cell-cell junctions in *Mer* ^*ts1*^ mutants using transmission electron microscopy (TEM). We observed electron dense cell-cell junctions at the CC-CC interface in control testis (Fig. 3F, arrows). Such electron-dense junctions between two CCs were consistently observed along the cell-cell interface. In contrast, we found that the cell-cell junction between two somatic cells was significantly weaker in *Mer* ^*ts1*^ mutants (Fig. 3G, arrows), and patches that lacked electron-dense junctional structures were frequently observed in *Mer* ^*ts1*^ mutants (Fig. 3G, double-headed blue arrows). Although its significance remains unclear, we frequently observed vesicular compartment resembling multivesicular bodies in *Mer* ^*ts1*^ mutant CCs (Fig. 3G, yellow arrowhead). The TEM analysis, combined with immunofluorescent staining analysis of multiple adherens junction markers, suggests that *Mer* is required for stabilization of cell-cell adhesion between CCs.

### *Mer* mutant CySCs are triggered to undergo tumorous overproliferation upon stimulation by the bone morphogenetic protein (BMP) ligand *Dpp*

A moderate increase in the number of CCs in the *Mer* mutant/RNAi testes suggests that *Mer* is required for suppressing CySC proliferation. Contact-dependent inhibition of proliferation is a mechanism that safe-guards against overproliferation of cells, making cells resistant to stimulation by mitogens. Therefore, defects in contact-dependent inhibition may not have a profound effect in the absence of mitogens.

**Figure 4:**
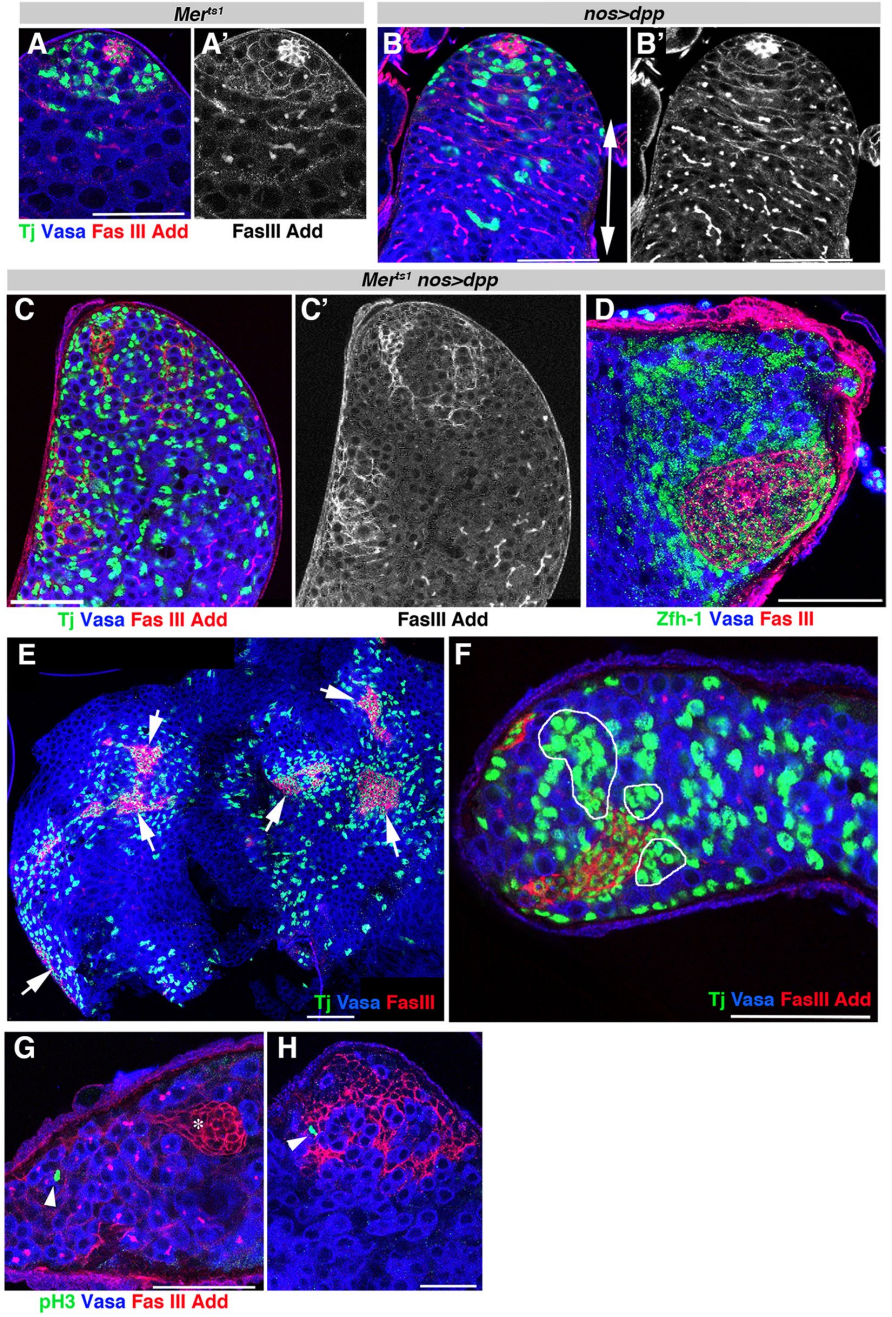
Ectopic expression of Dpp drives tumorous CySC/CC expansion in the *Mer* mutant. (**A**–**C**) The apical tip of the testis in *Mer* ^*ts1*^ (**A**), *nos* > *dpp* (**B**), and *Mer* ^*ts1*^ *; nos* > *dpp* (**C**) stained for Tj (Green), Vasa (Blue), Add, and Fas III (Red). Bar, 50 µm. (**D**) The apical tip of the testis from *Mer* ^*ts*1^; *nos* > *dpp* stained for Zfh-1 (Green), Vasa (Blue), and Fas III (Red). (**E**) The apical tip of the testis from *Mer* ^*ts1*^; *nos* > *dpp* stained for Tj (Green), Vasa (Blue), and Fas III (Red). Expansion of hub cells are shown by arrows. (**F**) The apical tip of the testis from *Mer* ^*ts1*^; *nos* > *dpp* stained for Tj (Green), Vasa (Blue), and Fas III (Red). Clusters of Tj^+^ CCs are indicated by white circles. (**G** and **H**) Examples of mitotic somatic cells [positive for phosphorylated histone H3 (pH3)] away from the hub (**G**) and within the expanded hub-like structure (**H**) in a *Mer* ^*ts1*^; *nos* > *dpp* testis. Hub (*).

We reasoned that, if *Mer* regulates CySC proliferation via the mechanism of contact inhibition, *Mer* mutant may not exhibit severe phenotypes unless stimulated by mitogens. Based on this idea, we examined a potential effect of ectopically expressing signaling ligands in *Mer* ^*ts1*^ mutant background. Dpp, Hedgehog (Hh), Delta (Dl), Wingless (Wg), or Spitz (Spi, an Egf ligand), which are known to be expressed in the *Drosophila* testicular niche^16, 28–33^, were expressed in wild type or *Mer* ^*ts1*^ mutant background (+; *nos-gal4* > *UAS-ligand* or *Mer* ^*ts1*^; *nos-gal4* > *UAS-ligand*) and its effect was examined. Expression of Hh, Dl, Wg or Spi did not cause any detectable defects in tissue architecture in wild type or *Mer* ^*ts1*^ background (Supplementary Figure S4). Overexpression of Dpp in wild type background led to expansion of SGs due to its known role to suppress differentiation (Fig. 4B double-headed arrow)^30–32, 34, 35^. However, overall architecture of the tissue was maintained with differentiation progressing along the apical-to-basal axis of the testis. In contrast, when Dpp overexpression was combined with *Mer* ^*ts1*^, massive proliferation of CySC/CCs were observed, leading to perturbed tissue architecture due to tumorous overproliferation of cells (Fig. 4C–H). The numbers of Tj^+^ CCs as well as Zfh-1^+^ CySCs increased dramatically in *Mer* ^*ts1*^ Dpp-expressing testes compared to Dpp-expressing testis in wild type background (Fig. 4C–F). We often observed large clusters of Tj^+^ CC cells, especially near the Fas III-positive hub–like clusters (Fig. 4F, circles), indicating that *Mer* ^*ts1*^ mutant CySCs overproliferated without coordinating with germ cells. Moreover, in *Mer* ^*ts1*^ Dpp-expressing testes, hub cells were enlarged (Fig. 4D, 48% of testes, N = 42), or multiple hub-like clusters were observed (Fig. 4C and E, 33% of testes, N = 42). These hub-like cells may be derived from transformation of CySCs/CCs to hub-like fate associated with expansion of CySC/CC pool^36^. In a *Mer* ^*ts1*^ mutant background, not only CySCs, but also CCs as well as hub-like cells, underwent cell division visualized by mitotic cell marker phosphor-histone H3 (Fig. 4G and H, 0 mitotic CCs/testis in *Mer* ^*ts1*^ control, N = 117 testes, 0.64 mitotic CCs/testis in *Mer* ^*ts1*^; *nos-gal4* > *UAS-Dpp*, N = 120 testes), whereas CCs or hub cells never underwent mitosis in a wild-type background^25, 36^ or in Dpp-overexpressing testis. These results indicate that *Mer* ^*ts1*^ mutant somatic cells are sensitive to proliferative stimuli due to expression of Dpp. Importantly, the fact that Dpp overexpression in wild type background does not cause CySC/CC expansion suggests that wild type CySCs/CCs are resistant to stimulation by Dpp.

### Dpp pathway in CySCs functions to promote CySC mitosis separately from its known function in germ cells

Based on the results described above, we hypothesized that CySCs/CCs are normally prevented from overproliferation by the function of *Mer*, and that Dpp has a mitogenic effect on CySCs/CCs. Dpp overexpression in wild type background would not lead to CySC/CC overproliferation because *Mer* prevents them from overproliferating. *Mer* mutant CySCs/CCs would not overproliferate on its own, either, because mitogenic stimulation (Dpp) is limited. Only when combined, however, ectopically expressed Dpp stimulates *Mer* mutant CySCs/CCs, leading to overproliferation.

This hypothesis postulates that Dpp has a mitogenic activity on CySCs/CCs. To test in which cell type Dpp signaling must be active to stimulate the proliferation of *Mer* ^*ts1*^ mutant CySCs/CCs, we first expressed a constitutively active form of Tkv (Tkv*), the receptor for Dpp, in germ cells of *Mer* mutant testes (*Mer* ^*ts1*^; *nos-gal4* > UAS-tkv*): Unlike the expression of the ligand (Dpp), which can be secreted and act in both autocrine and paracrine manners, the receptor (Tkv) would be confined within the cell in which the expression is driven. Expression of Tkv* in germ cells in combination with *Mer* ^*ts1*^ did not cause tumorous overproliferation, although a spermatogonial tumor was observed consistent with the role of Tkv in the germline to suppress differentiation (Supplementary Figure S5A, double-headed arrow)^30–32, 34, 35^. This result suggests that Dpp-Tkv pathway does not operate in germ cells to stimulate CySC/CC proliferation. Instead, this result suggests that Dpp-Tkv pathway operates in CySC/CCs to stimulate their proliferation.

Strikingly, expression of Tkv* in the CySC lineage (*c587-gal4* > tkv*) was sufficient to induce CySC overproliferation even in the wild type background (Supplementary Figure S5B,C). This result has two important implications. First, it supports the idea that Dpp-Tkv signaling in CySC lineage functions to promote their proliferation. Second, the fact that overexpression of Tkv* is sufficient to drive CySC/CC proliferation even without *Mer* mutation indicates that *Mer* functions downstream of Dpp secretion/reception but upstream of Tkv activation. Taken together, these results suggest that Dpp functions as a mitogen for CySC lineage, and CySC proliferation is controlled at least at two levels: limiting available Dpp and *Mer*-dependent mechanism that makes CySCs resistant to stimulation by Dpp.

### *Mer* functions with E-cadherin in regulating CC number

*Nf2/Mer* mediates contact-dependent inhibition of proliferation through regulation of adherens junction^22, 23^. The results described above (Fig. 3) are consistent with *Mer*’s role in regulating adherens junction. To gain further insights into the relationship between *Mer* and E-cadherin in suppressing proliferation of CySCs/CCs, we examined potential genetic interactions between these two genes in regulating CC number.

**Figure 5:**
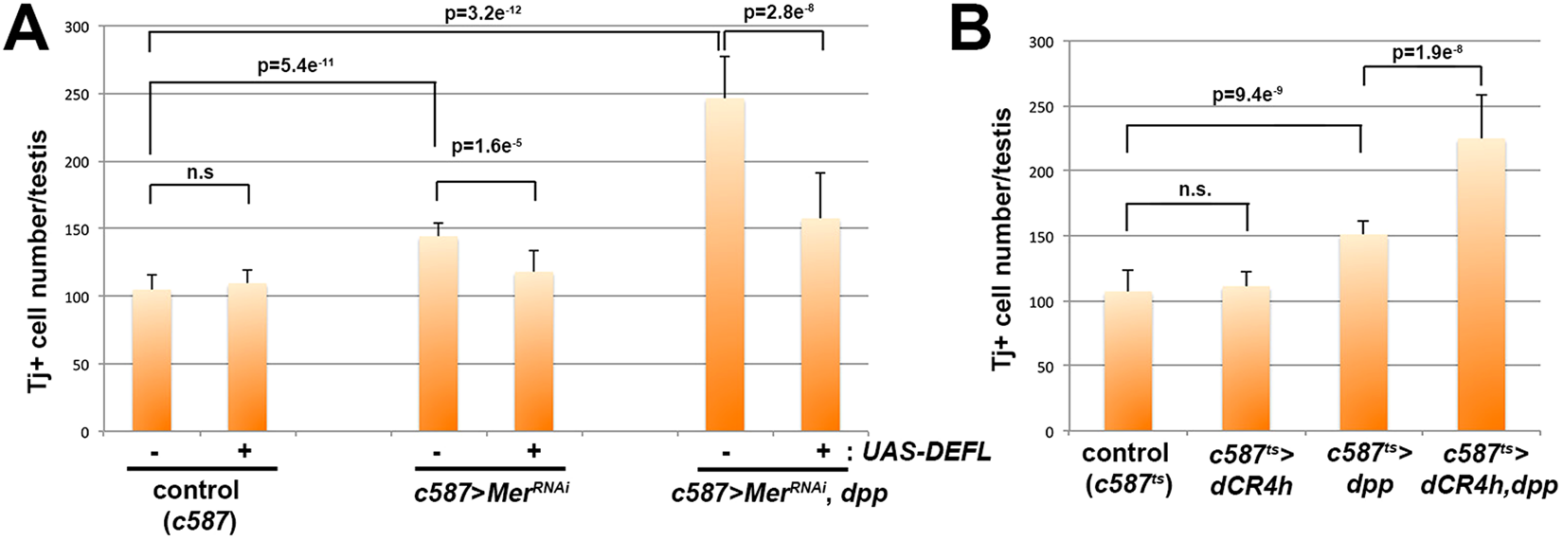
E-cadherin functionally interacts with *Mer* and Dpp in regulation of CySC/CC proliferation. (**A**) Tj^+^ CC number in control, *Mer* ^*RNAi*^, and *Mer* ^*RNAi*^; *nos* > *dpp* testes in the presence or absence of E-cadherin overexpression. N = 15 testes for each data point. (**B**) Tj^+^ CC number in control, *c587* > *dCR4h, c587* > *dpp*, and *c587* > *dCR4h, dpp* testes. *c587-gal4* was combined with *tub-gal80* ^*ts*^, and expression was induced upon eclosion by shifting young males from 18 °C to 29 °C for 7 days. N ≥ 15 testes for each data point. *p* values were obtained using the Student’s *t* test (two-tailed).

We first tested whether expression of wild-type E-cadherin (*UAS-DEFL*) might be able to suppress the increase in Tj^+^ CC number due to *Mer* ^*RNAi*^. As described above, *Mer* ^*RNAi*^ in the CySC lineage caused a moderate but significant increase in Tj^+^ CC number. Expression of E-cadherin in *Mer* ^*RNAi*^ background reduced Tj^+^ CC number significantly (Fig. 5A), suggesting that the phenotypes of *Mer* ^*RNAi*^ comes at least partly from destabilized adherens junction. We next tested whether expression of E-cadherin can suppress the increase in Tj^+^ CC number due to combinatory effect of *Mer* ^*RNAi*^ and ectopic Dpp expression. Similar to the case of *Mer* ^*ts1*^, the increase in Tj^+^ CC number in *Mer* ^*RNAi*^ testis was further enhanced by co-expression of Dpp (Fig. 5A, ~100 Tj^+^ CCs in *c587-gal4* > *UAS-Mer* ^*RNAi*^ compared to ~250 Tj^+^ CCs in *c587-gal4* > *UAS-Mer* ^*RNAi*^ *, UAS-dpp*). Such increase in Tj^+^ CC number was dramatically suppressed by co-expression of E-cadherin (Fig. 5A, ~150 Tj^+^ CCs), suggesting that increased stability of adherens junction can rescue defects caused by loss of *Mer* function.

If an increase in Tj^+^ CC number in *Mer* ^*RNAi*^ is due to, at least in part, destabilized adherens junctions, weakening adherens junctions by overexpressing a dominant-negative E-cadherin mutant (*UAS-dCR4h*, an E-cadherin mutant that lacks the extracellular domain^37^) might be sufficient to make CySCs/CCs sensitive to Dpp overexpression. Indeed, combined expression of Dpp and dCR4h significantly increased Tj^+^ CC number, even in the absence of the *Mer* mutation or RNAi (Fig. 5B), demonstrating that CCs with weakened cell-cell junctions are more susceptible to stimulation by Dpp. Taken together, these data support the model, in which *Mer* functions to stabilize adherens junctions, which in turn suppresses excess proliferation of CySCs.

### *Mer* is required to prevent CySC overproliferation in the absence of germ cells

**Figure 6:**
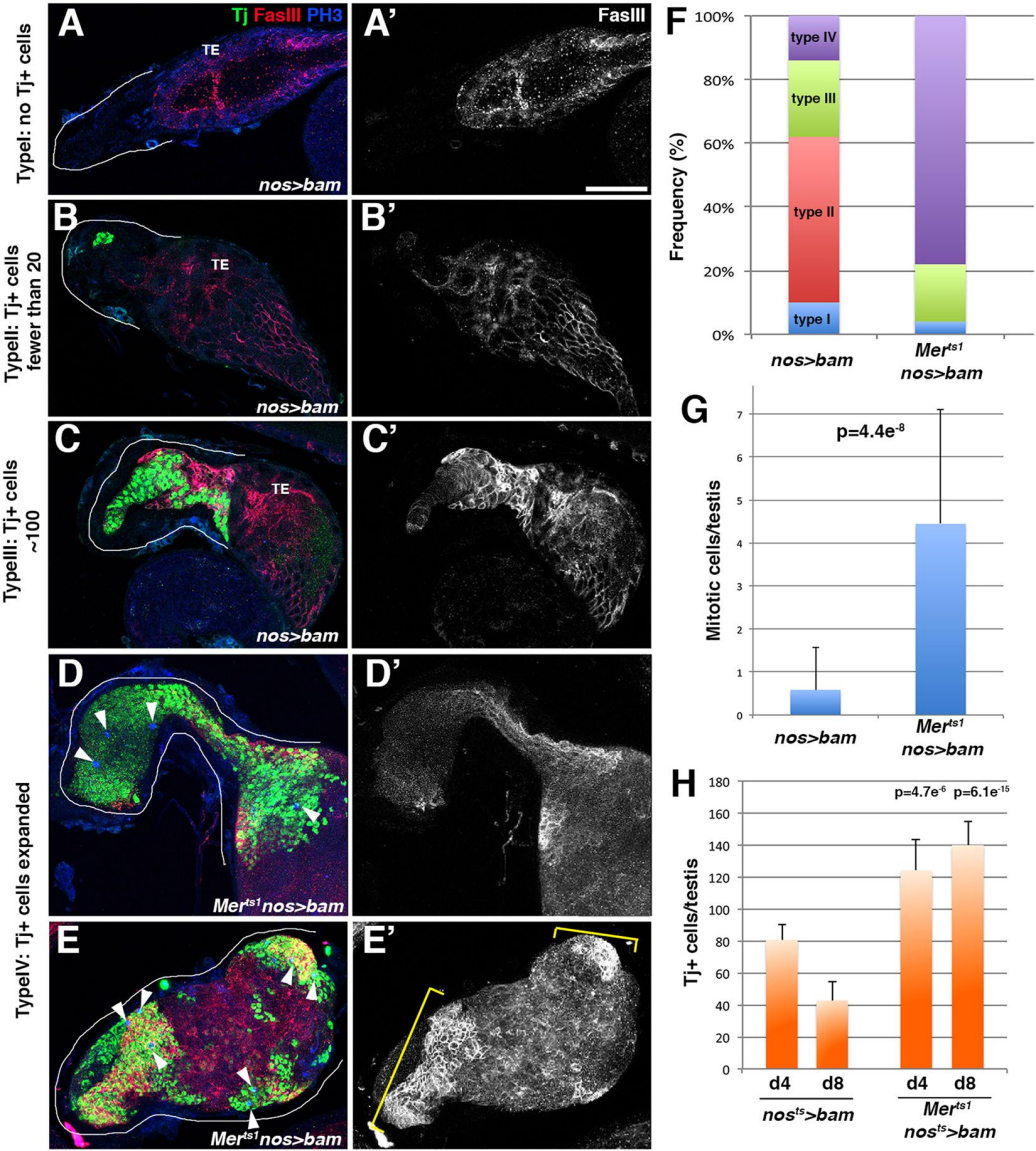
*Mer* is required to prevent CySC/CC overproliferation in the germ cell-depleted testis. (**A**–**C**) The apical tip of the testis from *nos* > *bam* stained for Tj, FasIII, and phosphorylated histone H3 (pH3). The testis apical region is marked by white lines. Terminal epithelium (TE). Bar, 50 µm. (**D** and **E**) The apical tip of the testis from *Mer* ^*ts1*^ *nos* > *bam*. Overproliferated Tj^+^ cells stained for FasIII (D’ and E’). Arrowheads indicate mitotic somatic cells. (**F**) Summary of the testis phenotype upon depletion of germ cells in the wild-type or *Mer* ^*ts1*^ background. Types I–IV correspond to the designations in A–E. n ≥ 27 testes for each data point. (**G**) The mitotic index in testes expressing *bam* in a wild-type or *Mer* ^*ts1*^ mutant background. N = 30 testes for each data point. (**H**) The number of Tj^+^ cells in wild-type and *Mer* ^*ts1*^ background after induction of germ cell loss by expression of *bam* upon eclosion. N ≥ 10 testes for each data point. *p* values were obtained using the Student’s *t* test (two-tailed) by comparing to control at corresponding time points.

While the sensitivity of *Mer* mutant CySCs/CCs to Dpp overexpression reveals that they are defective in preventing overproliferation, Dpp is not normally expressed broadly in the testis^30^. Thus, our experimental model described above (ectopic expression of Dpp) might be somewhat artificial, although it highlights the defective nature of *Mer* mutant in preventing CySC proliferation. To further address the role of *Mer* in preventing excess proliferation of CySCs/CCs in coordination with germ cells, we examined the effect of germ cell depletion on CySCs/CCs in *Mer* mutant. Bam is a master regulator of differentiation^38^, and its expression in germ cells (*nos-gal4* > *UAS-bam*) results in complete loss of germ cells by the time of eclosion. In a wild-type background, *bam*-induced germ cell depletion was associated with underdeveloped testicular structure, frequently containing few or no Tj^+^ cells (Fig. 6A–C, F, type I and II testes). Compared to the wild-type testis that has an average of ~100 Tj^+^ cells (Fig. 2C), *bam*-expressing testes contain much fewer Tj^+^ cells, indicating that CySCs responded to the lack of germ cells and ceased proliferation during development. In stark contrast to the wild-type testis depleted of germ cells, the *Mer* ^*ts1*^ mutant testes depleted of germ cells (*Mer* ^*ts1*^; *nos-gal4* > *UAS-bam*) often exhibited overproliferation of Tj^+^ CCs (Fig. 6D–F, type III and IV). CCs in such testes maintained proliferation, as assessed by the presence of phosphorylated histone H3 (pH3) (Fig. 6D and E, arrowheads, and 6G). Similar results were obtained even when *bam* was turned on after eclosion by temperature shift (*Mer* ^*ts1*^; *nos-gal4ΔVP16, tub-gal80* ^*ts*^ > *UAS-bam*)(Fig. 6H), suggesting that *Mer*-dependent prevention of CySC/CC proliferation in response to germ cell depletion operates during development as well as adult tissue homeostasis.

Taken together, these results suggest that *Mer* is required for suppressing CySC proliferation when not accompanied by germ cell proliferation. We propose that *Mer* prevents CySC overproliferation via a mechanism similar to contact-dependent inhibition of proliferation, wherein ‘overcrowding’, or confluency, of CySCs/CCs in relation to germ cells suppresses CySC proliferation.

## Discussion

Despite its paramount importance in tissue development and maintenance, the mechanisms by which multiple stem cell lineages coordinate proliferation remain poorly understood. Does one lineage have instructive or permissive roles over the other lineage(s)? Do they crosstalk to coordinate proliferation? How do the tissues sense the correct number and/or ratio of cells among multiple lineages to maintain the functional tissue? Our previous study using overexpression of Cdc25, a major mitotic regulator, suggested that CySCs have a permissive (but not instructive) role over GSC proliferation^7^. However, how CySC proliferation might be coordinated with GSC proliferation remains unclear.

The present study showed that *Mer* is an important regulator of CySC division. We propose *Mer* functions in a manner reminiscent of *Nf2*’s role in contact-dependent inhibition of proliferation^9, 12, 23^. In the absence of *Mer* function, the number of CCs mildly increased in relation to germ cells, leading to extra CCs. Such a phenotype could be easily missed, as *Mer* mutant testes show only a slight increase in CC number and maintain an overall normal tissue architecture. However, *Mer’*s requirement for the regulation of CySC proliferation was revealed in a sensitized background. First, when cell proliferation was stimulated by ectopic expression of Dpp, *Mer* mutant CySCs/CCs underwent tumorous overproliferation. Importantly, Dpp overexpression in a wild-type background does not lead to tumorous overgrowth of CySCs/CCs, suggesting that *Mer* plays a role to make CySCs/CCs resistant to mitogenic stimulation. Second, *Mer* mutant CySCs/CCs continue to proliferate in the absence of germ cells due to overexpression of Bam, a master regulator of differentiation.

By drawing a parallel between the established role of *Nf2* in contact inhibition and that of *Mer* in preventing CySC/CC overproliferation, we propose that ‘confluency’ of one lineage (e.g. CySC lineage) with respect to the other lineage (e.g. germline) serves as a mechanism to coordinate the proliferation of two lineages in a given tissue. In this scenario, CySCs proliferate until they and their progeny occupy the surface of germline, reaching ‘confluency’, at which point contact inhibition mechanism mediated by *Mer* and adherens junctions halts CySC division. Once GSCs divide, it would increase ‘substrate surface’ (i.e. surface generated by production of more germ cells) on which CySC lineage can proliferate, until their progeny reach to confluency again. In this manner, GSC and CySC divisions would balance their proliferation to maintain correct ratio of cell numbers. Taken together, our study illuminates the mechanism by which two distinct stem cell populations within a tissue coordinate their proliferation to maintain tissue homeostasis, and provide insights into how contact inhibition may operate in tissues in the *in vivo* context.

## Materials and Methods

### Fly Husbandry and Strains

All fly stocks were raised in standard Bloomington medium. The following fly stocks were used: *Mer* ^*ts1*^ ^39^; a gift from Ilan Davis), *c587-gal4* ^40^, *nos-gal4* ^41^, *UAS-Mer* ^*RNAi*^ (GD1484 from the Vienna *Drosophila* Research Center), UAS-tkv* ^42^; a gift from Ting Xie), UAS-Bam^43^ a gift from Dennis McKearin), *UAS-DEFL* ^37^; a gift from Hiroki Oda), *hs-FLP; act* > *stop* > *gal4 UAS-GFP* ^44^; a gift from Yu Cai), *UAS-dpp, UAS-yki, UAS-ykiS168A*, *socs36E* ^*EY06665*^, and Df(2L)Exel7070 (obtained from the Bloomington Stock Center). These strains are described in Flybase (http://flybase.org). *nos-gal4* without VP16^45^ is denoted as *nos-gal4ΔVP16* to distinguish it from *nos-gal4-VP16* that was generated by^41^, which has been often referred to as *nos-gal4*. *nos-gal4ΔVP16* was combined with *tubulin-gal80* ^*ts*^ to achieve temperature-dependent, temporal control of *UAS-bam* expression.

*Mer* ^*ts1*^ flies were raised at 18 °C and shifted to 29 °C upon eclosion for 2–3 days before analysis. Expression of Dpp or DEFL under the *c587-gal4* driver was performed by raising flies at 18 °C to avoid lethality during development and shifted to 25 °C upon eclosion for 2–3 days before analysis. Other fly crosses were performed at 25 °C. Control experiments were conducted with matching temperature-shift schemes.

### Immunofluorescent Staining

Immunofluorescent staining was performed as described previously^46^. Briefly, testes were dissected in phosphate-buffered saline (PBS) and fixed in 4% formaldehyde in PBS for 30–60 minutes. Next, testes were washed in PBST (PBS +0.1% Tween 20) for at least 30 minutes, followed by incubation with primary antibody in 3% bovine serum albumin (BSA) in PBST at 4 °C overnight. Samples were washed for 60 minutes (three times for 20 minutes each) in PBST, incubated with secondary antibody in 3% BSA in PBST at 4 °C overnight, and then washed for 60 minutes (three times for 20 minutes each) in PBST. Samples were then mounted using VECTASHIELD with 4′,6-diamidino-2-phenylindole (DAPI). The primary antibodies used were as follows: mouse anti-adducin-like [1:20, developed by H. D. Lipshitz and obtained from the Developmental Studies Hybridoma Bank (DSHB)], anti-Fasciclin III (1:100, developed by C. Goodman and obtained from DSHB), anti-βPS (1:20, developed by D. Brower and obtained from DSHB), anti-E-cadherin (1:20, developed by T. Uemura and obtained from DSHB), rabbit anti-Thr3-phosphorylated histone H3 (1:200; Upstate), rat anti-Vasa (1:40; developed by A. Spradling and D. Williams, and obtained from DSHB), rabbit anti-Zfh-1 (1:4000; a gift from Ruth Lehmann), guinea pig anti-Tj (1:400, a gift from Dorothea Godt), and guinea pig anti-*Mer* (1:2000, a gift from Rich Fehon). AlexaFluor-conjugated secondary antibodies were used at a dilution of 1:200. Images were taken using a Leica TCS SP5 or SP8 confocal microscope with a 63 × oil immersion objective (NA = 1.4) and processed using Adobe Photoshop software.

### Transmission Electron Microscopy

*Drosophila* testes were dissected in 1 × PBS and fixed in 2% glutaraldehyde/2% paraformaldehyde (EM grade) in 0.1 M cacodylate (pH 7.4) for 5 minutes at room temperature. This step was followed by an additional 25-minute fixation on ice. The tissue was rinsed three times for 10 minutes each in cacodylate buffer and then post-fixed for 30 minutes in 2% osmium tetroxide in the same buffer on ice. Next, the samples were rinsed in double-distilled water and then stained *en bloc* for 1 hour in aqueous 7% uranyl acetate. The samples were then dehydrated in increasing concentrations of ethanol, treated with propylene oxide, and embedded in Epon epoxy resin. Semi-thin sections were stained with toluidine blue for tissue identification. Selected regions of interest were serially sectioned (70-nm thickness) and mounted on Formvar/carbon-coated slotted grids. The grids were post-stained with uranyl acetate and lead citrate, and samples were examined using a Philips CM100 electron microscope at 60 kV. Images were recorded digitally using a Hamamatsu ORCA-HR digital camera system, which was operated using AMTsoftware (Advanced Microscopy Techniques Corp., Danvers, MA).

## Electronic supplementary material

Supplementary Figures
