# Supplementary material for: Merlin is required for coordinating proliferation of two stem cell lineages in the Drosophila testis
Source: Sci Rep. 2017 May 31;7:2502. doi: 10.1038/s41598-017-02768-z (PMC5451480; doi:10.1038/s41598-017-02768-z)
Supplement: Supplementary file 1 — Supplementary Figures [file 41598_2017_2768_MOESM1_ESM.pdf]

Supplementary Information

Merlin is required for coordinating proliferation of two stem cell lineages in the *Drosophila* testis

Mayu Inaba,[1, 2, 3, **] Dorothy R. Sorenson,[2] Matt Kortus,[2] Viktoria Salzmann,[1, 2] and Yukiko M. Yamashita[1, 2, 3, *]

[1]Life Sciences Institute, Center for Stem Cell Biology, [2]Department of Cell and Developmental Biology, School of Medicine, [3]Howard Hughes Medical Institute, University of Michigan, Ann Arbor,

*To whom correspondence should be addressed: Yukiko M. Yamashita, 210 Washtenaw Ave, Rm5403, University of Michigan, Ann Arbor, MI 48109, USA
Tel: 734-615-8508; Email: yukikomy@umich.edu

**Present address: 263 Farmington Ave. E6053, Department of Cell Biology, UConn Health, Farmington, CT 06030, USA

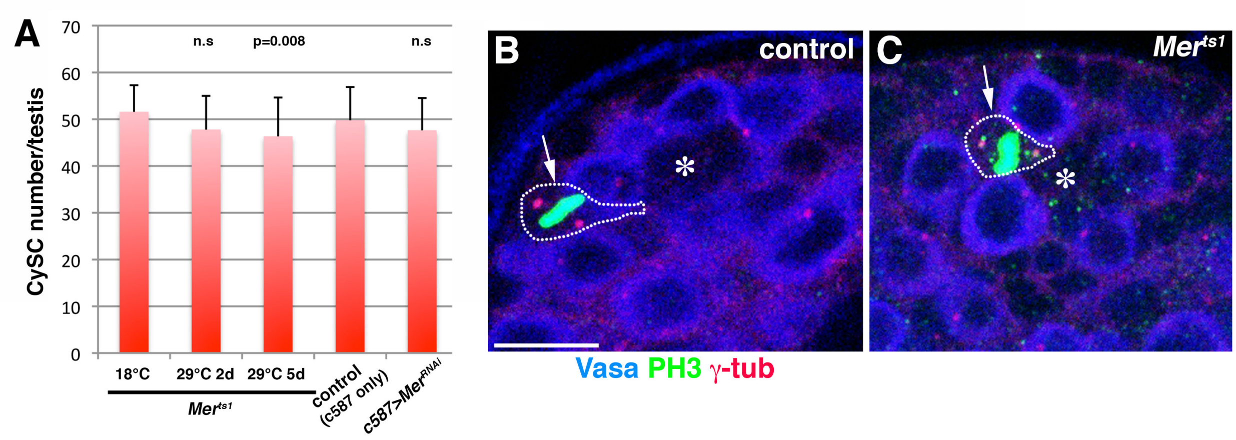

Figure S1. The effect of *Mer* mutation/knockdown on CySCs.

A) The number of Zfh-1⁺ cells/testis in *Mer*^{ts1} mutant and *c587-gal4>UAS-Mer*^{RNAi} testes. B, C) Examples of mitotic CySCs (arrows) in control (B) and *Mer*^{ts1} mutant (C, 29°C) testes, where CySC mitoses were limited to next to the hub. Bar 10µm.

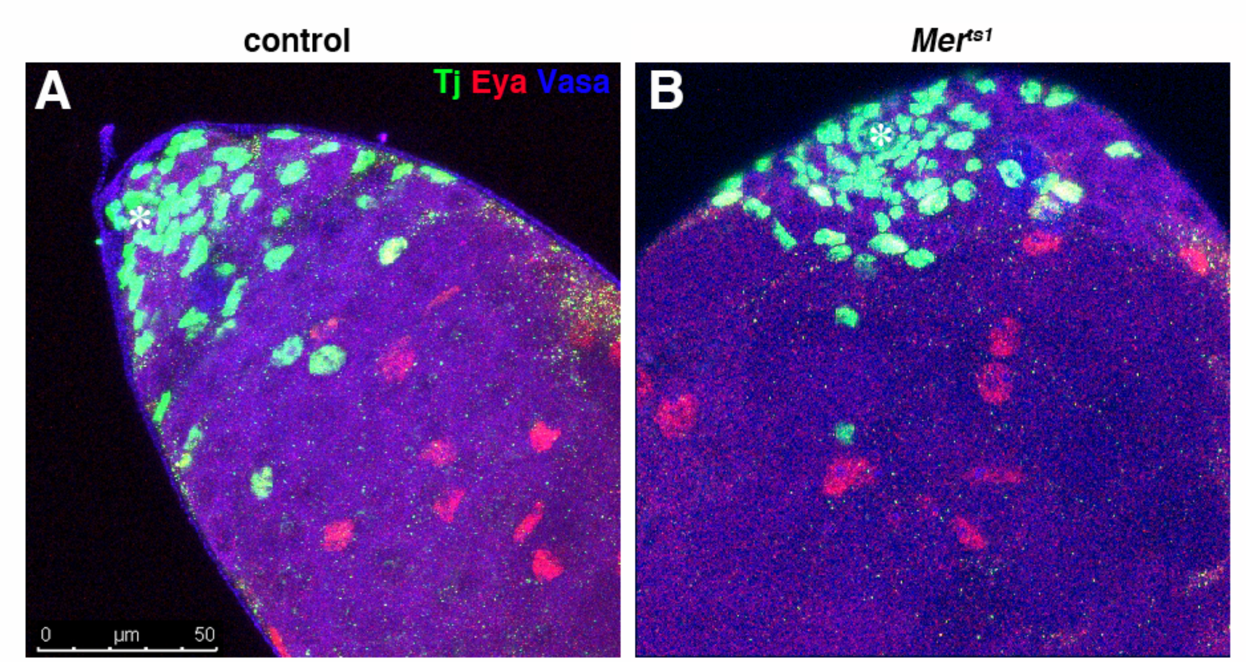

Figure S2. ***Mer* mutant cyst cells differentiate normally.**
Control (A) and *Mer*^{ts1} (B) testes stained with anti-Tj, -Eya, and -Vasa antibodies, demonstrating that CCs differentiate normally into Eya-positive cells.

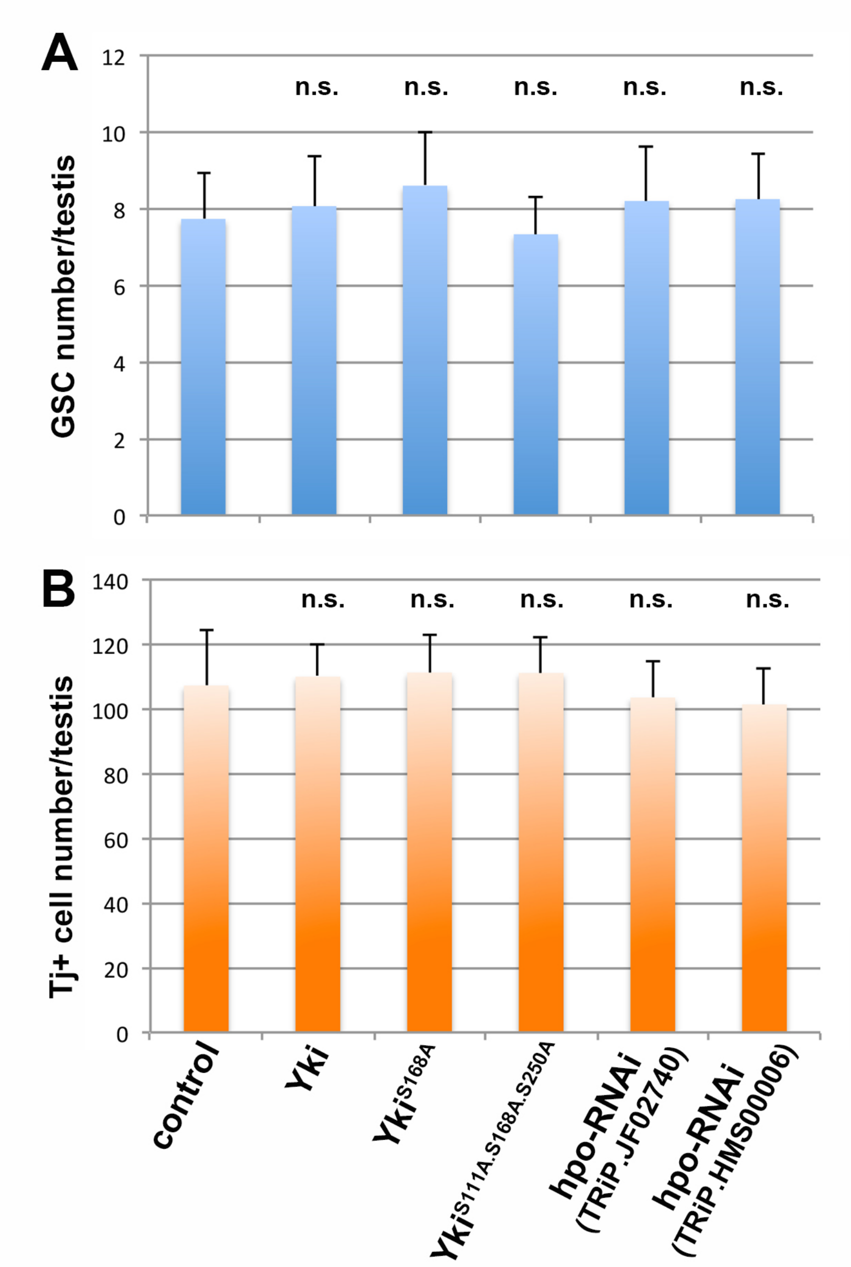

Figure S3. Neither Yki nor Hippo regulates Tj+ cyst cell number.

Yki, its nonphosphorylatable forms, and two independent RNAi constructs were expressed under the control of *c587-gal4* and combined with *tubulin-gal80*^{ts}. The flies were cultured at 18°C until eclosion to avoid lethality during development, then transferred to 29°C for 7 days to allow transgene expression before analysis. Expression of neither *yki* nor *hpo*^{RNAi} affected either GSC number (A) or Tj^{+} cyst cell number (B).

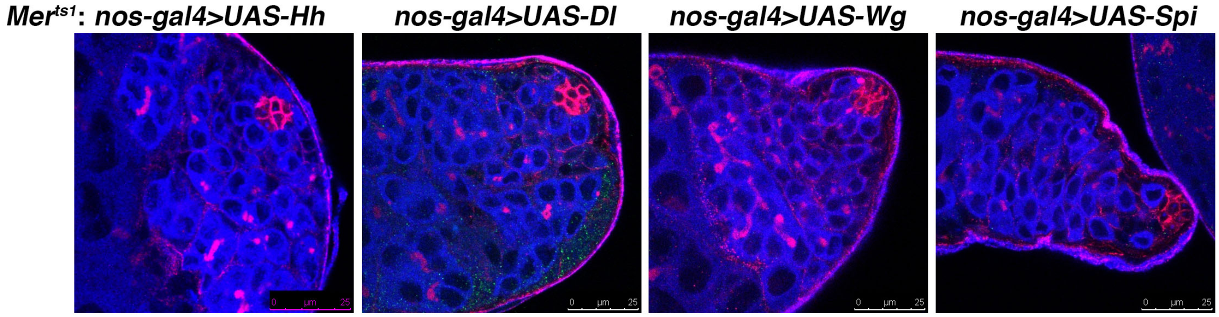

Figure S4. Unchanged overall tissue architecture of testes upon overexpression of *Hh, Dl, Wg* or *Spi* in *Mer^{ts1}* mutant background.
Blue: Vasa, Red: FasIII and Add

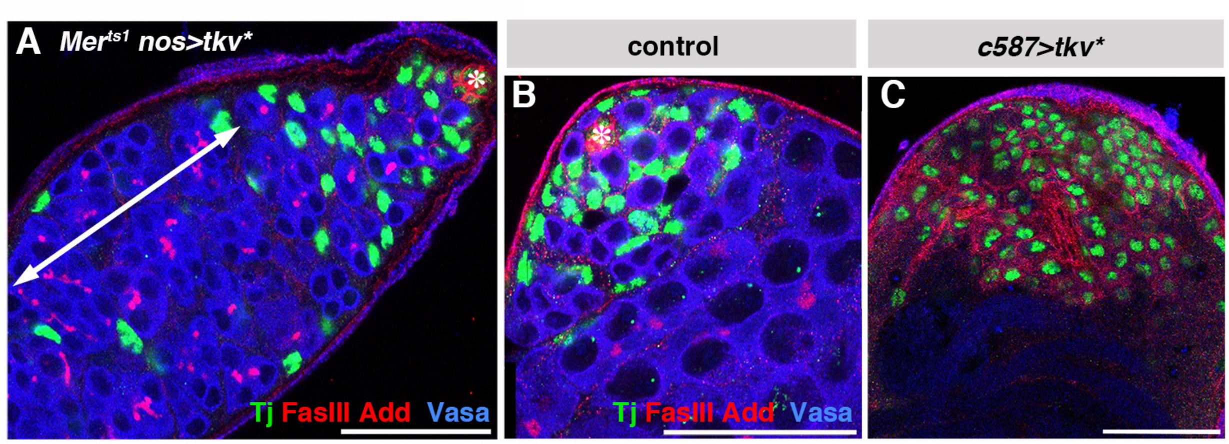

Figure S5. Dpp-Tkv signaling in somatic CySCs/CCs regulates CySC proliferation.

A) *Mer^{ts1} nos-gal4>UAS-tkv** shows spermatogonial overproliferation (double-headed arrow) but not tumorous expansion of CCs.

B, C) Apical tip of the testes in control (B) and *c587-gal4>UAS-tkv** (C). Expression of Tkv* is sufficient to cause CC overproliferation.
